# Interpretation Challenges in Combined Botulinum Toxin A and Profhilo Therapy: Letter to the Editor

**DOI:** 10.1002/hsr2.72798

**Published:** 2026-07-13

**Authors:** Yudai Kaneda

**Affiliations:** ^1^ Clinical Training Center, Jyoban Hospital of Tokiwa Foundation Fukushima Japan; ^2^ Health in Humanitarian Crises, London School of Hygiene & Tropical Medicine London UK

## Author Contributions

**Yudai Kaneda:** conceptualization, writing – original draft.

## Funding

The author has nothing to report.

## Conflicts of Interest

The author declares no conflicts of interest.

## Transplant Statement

Yudai Kaneda affirms that this manuscript is an honest, accurate, and transparent account of the research reported, that no important aspects of the research have been omitted, and that any differences from the planned research (and registration, if relevant) are explained by Yudai Kaneda.

Editior,

I read with great interest the article by Saffarian et al. on the combined use of botulinum toxin A (BTX‐A) and Profhilo for upper face rejuvenation [1]. The study found that both the BTX‐A–only group and the BTX‐A plus Profhilo group showed significant wrinkle improvement, without a significant between‐group difference or added benefit from Profhilo. The authors should be commended for conducting a randomized clinical trial addressing an increasingly relevant question in aesthetic dermatology. However, several methodological and clinical issues merit clarification, as they may substantially influence the interpretation of the findings.

First, although the authors state that skin elasticity was measured, neither the methodology nor the corresponding quantitative data are clearly presented, as elasticity results are not shown in the tables or figures. This is notable given that dermal thickness and hypodermal thickness are explicitly reported in Table 2, while elasticity, arguably the primary target of Profhilo, remains unquantified. As Profhilo is expected to exert its principal effect on skin quality through bio‐remodeling mechanisms, the lack of transparent reporting limits evaluation of biological plausibility [2].

Second, inconsistencies in reporting raise concerns. While 20 participants are described (10 per group), certain analyses appear to include fewer cases (e.g., *n* = 8 in figures 3, 4, and 5), without explanation. In addition, the definition of the “volume” metric and the interpretation of the confidence intervals reported alongside *p*‐values are unclear. Furthermore, Figure 3 appears visually unclear and difficult to interpret at a glance, potentially obscuring the primary clinical message. Clear reporting of analyzed populations and outcome definitions is fundamental for proper interpretation and reproducibility.

Third, generalizability may be limited by the relatively young (mean age ~ 30 years) and male‐dominant study population, which differs from typical aesthetic practice, where middle‐aged female patients with more established static wrinkles represent the majority [3]. Furthermore, variability in dosing (4–16 units per injection point across 7–10 points) and the use of an intradermal injection technique may limit comparability with established protocols. Most importantly, the timing of outcome assessment differs between groups. The control group was evaluated approximately 3 months after BTX‐A injection, whereas the combination group was assessed 3 months after the final Profhilo session, resulting in a longer interval from BTX‐A administration. Given that BTX‐A effects typically decline after approximately 3–5 months in aesthetic indications, this temporal inconsistency may introduce bias [4]. In addition, the total BTX‐A dose per participant is not reported despite wide dosing variability, which may further confound results.

In light of these issues, a brief clarification of elasticity data, endpoint‐specific sample sizes, exclusions and reasons, volume measurement methods, and confidence interval definitions, along with sensitivity analyses adjusted for time since BTX‐A injection and exploratory stratification by dose and baseline severity, would substantially improve interpretability. Such clarification aligns with fundamental CONSORT principles regarding participant flow, analyzed populations, and transparent reporting of effect estimates [5].

A response from the authors addressing these points would be highly appreciated, particularly regarding the implications of incomplete outcome reporting, inconsistencies in analyzed populations, and differences in the timing of outcome assessment in this study.

## Data Availability

Data sharing not applicable to this article as no datasets were generated or analyzed during the current study.
